# Fine Mapping and Candidate Gene Identification for Wax Biosynthesis Locus, *BoWax1* in *Brassica oleracea* L. var. *capitata*

**DOI:** 10.3389/fpls.2018.00309

**Published:** 2018-04-30

**Authors:** Dongming Liu, Xin Dong, Zezhou Liu, Jun Tang, Mu Zhuang, Yangyong Zhang, Honghao Lv, Yumei Liu, Zhansheng Li, Zhiyuan Fang, Limei Yang

**Affiliations:** ^1^Key Laboratory of Biology and Genetic Improvement of Horticultural Crops, Ministry of Agriculture, The Institute of Vegetables and Flowers, Chinese Academy of Agricultural Sciences, Beijing, China; ^2^Open Key Laboratory of Horticultural Plant Physiology and Genetic Improvement, High School of Henan Province, College of Horticulture, Henan Agricultural University, Zhengzhou, China; ^3^Institute of Horticulture, Henan Academy of Agricultural Sciences, Zhengzhou, China

**Keywords:** cabbage, glossy green mutant, inheritance, gene mapping, candidate gene

## Abstract

Cuticular waxes play important roles in plant protection against various biotic and abiotic environmental stresses. The cuticular wax covering gives normal cabbage a glaucous appearance, but the appearance of waxless mutant is glossy green. Based on the present study, inheritance of the glossy green character of mutant HUAYOU2 follows a simple recessive pattern. Genetic analysis of an F_2_ population comprising 808 recessive individuals derived from HUAYOU2 (P_1_, maternal parent) and M36 (P_2_, paternal parent) revealed that a single recessive locus, *BoWax1* (*Brassica oleracea Wax 1*), controls glossy green trait in *B*. *oleracea*. This locus was mapped to a region of 158.5 kb on chromosome C01. Based on nucleotide sequence analysis, *Bol013612* was identified as the candidate gene for *BoWax1*. Sequencing results demonstrated that there is a deletion mutation of two nucleotides in the cDNA of *Bol013612* of HUAYOU2, which may account for its glossy green trait. These results lay the foundation for functional analysis of *BoWax1* and may accelerate research on wax metabolism in cabbage.

## Introduction

The aerial epidermis of terrestrial plants is coated with a cuticle that consists of cutin and cuticular wax (Jetter et al., 2007). Cutin is composed of modified fatty acids and provides mechanical strength to the surface layer (Kerstiens, 1996; Beisson et al., 2012) The cuticular wax, which embeds in and covers the cutin polymer, is composed of very long chain fatty acids (VLCFAs) and their derivatives, including primary and secondary alcohols, alkanes, aldehydes, ketones, and wax esters (Rashotte et al., 1997; Lee and Suh, 2015). Together with cutin, cuticular wax plays an important role in the protection of plants from damage caused by environmental stresses, such as water deficiency (Kerstiens, 1996; Mawlong et al., 2015), insect attacks (Stork, 1980), pathogen infection (Wang et al., 2006), and UV irradiance (Shepherd and Wynne Griffiths, 2006).

Wax composition and content can vary according to species, ontogeny, and environment (Jenks and Ashworth, 1999; Laila et al., 2016). As noted previously, the major components of wax are VLCFAs and their derivatives, including alkanes, aldehydes, primary and secondary alcohols, ketones, and esters (Riederer and Müller, 2006; Bernard and Joubès, 2013). C16 and C18 acyl-CoAs are precursors in the biosynthesis of C20–C34 VLCFA-CoAs, which are formed by the addition of C2 units derived from malonyl-CoA by the enzymes of the fatty acid elongase (FAE) complex in the endoplasmic reticulum (ER) (Kunst and Samuels, 2009; Li-Beisson et al., 2013). The FAE complex consists of β-ketoacyl-CoA synthase (KCS), β-ketoacyl-CoA reductase (KCR), 3-hydroxyacyl-CoA dehydratase (HCD), and *trans*-2,3-enoyl-CoA reductase (ECR), The FAE complex catalyzes four consecutive enzymatic reactions, resulting in a two-carbon extension of the acyl chain during each elongation cycle (Kunst and Samuels, 2009; Li-Beisson et al., 2013). The produced VLCFA-CoAs produced by the FAE complex are modified by two distinct pathways: an alkane-forming pathway and an alcohol-forming pathway. Most of the wax components, including aldehydes, alkanes, secondary alcohols, and ketones, are biosynthesized in the alkane-forming pathway, while the products of the alcohol-forming pathway, including primary alcohols and wax esters, account for only 10–15% of the total wax in *Arabidopsis* stems (Rowland et al., 2006).

Many genes associated with cuticular wax metabolism and its regulation in *Arabidopsis* and other plants have been identified with the help of waxless mutants. These genes include the FAE-related enzymes *KCS1, KCS2, KCS6, KCS9, KCS20*, and *KCR1* (Millar and Kunst, 1997; Millar et al., 1999; Todd et al., 1999; Fiebig et al., 2000; Pruitt et al., 2000; Hooker et al., 2002; Beaudoin and Kunst, 2009; Franke et al., 2009; Lee et al., 2009; Kim et al., 2013) and enzymes involved in the modification of VLCFA-CoAs, such as *CER1, CER3, MAH1, CER4*, and *WSD1* (Rowland et al., 2006, 2007; Greer et al., 2007; Li et al., 2008; Bourdenx et al., 2011), which play various roles in wax biosynthesis. *CER4* encodes an alcohol-forming fatty acyl-CoA reductase (FAR) that was reported to be responsible for the synthesis of primary alcohols in the epidermis of aerial tissues and in roots (Rowland et al., 2006).

Several genes implicated in wax biosynthesis of *Brassica* species have been reported in the recent studies. For example, the *BrWax1* gene in *Brassica rapa* was mapped to linkage group A01, and *Bra013809* was predicted to be the candidate gene for *BrWax1* (Zhang et al., 2013). The *BnaA.GL* gene in *Brassica napus* was finely mapped to a linkage group close to the end of chromosome A9 (Pu et al., 2013). In cabbage, the *BoGL1* gene was delimited to the end of chromosome C08 by a flanking marker, SSRC08–76, at a genetic distance of 0.2 cM (Liu D.et al., 2017). Although several genes in the alkane-forming pathway in *Brassica* species were isolated (Zhang et al., 2013; Liu Z. et al., 2017), no study concerning primary alcohol-forming pathway genes has been reported. In the present study, the *BoWax1* gene was mapped to chromosome C01, and *Bol013612* was identified as the candidate *BoWax1* gene. Based on the lower expression level of *Bol013612* and the differences in its sequence in the glossy green mutant HUAYOU2, the *Bol013612* gene was predicted to participate in wax biosynthesis in the plant through the alcohol-forming pathway. These results provide insight into the elucidation of the alcohol-forming pathway and the molecular mechanism of wax biosynthesis in *Brassica* species.

## Materials and Methods

### Plant Materials

The plant materials in this study were provided by our team named the Cabbage and Broccoli Research Group, Institute of Vegetables and Flowers (IVF), Chinese Academy of Agricultural Sciences (CAAS) and were grown in the greenhouse in Changping (39°54′ N, 116°13′ E, Beijing, China).

Five glossy green cabbage mutants have been reported previously (Tang et al., 2015). HUAYOU2 is also a cabbage mutant with a glossy green appearance. The mutant plant HUAYOU2 was initially discovered from the highly inbred line HUA2, which was used as the wild type (WT) line in this research. HUAYOU2 (P_1_, maternal parent) was crossed with M36 (P_2_, paternal parent), an inbred line of Chinese kale (*Brassica oleracea* var. *alboglabra*) with a glaucous appearance, to generate the F_1_, F_2_, BC_1_P_1_, and BC_1_P_2_ populations for inheritance and mapping studies. The BC_1_P_1_ and BC_1_P_2_ populations were created by backcrossing the F_1_ plant (maternal parent) with HUAYOU2 (paternal parent) and M36 (paternal parent), respectively. The same F_1_ plant was used as the female parent in the backcrosses and was also used to obtain the F_2_ population.

### SEM Analysis

Scanning electron microscopy (SEM) was employed to study the surfaces of the leaves of HUAYOU2 and WT plants. Fresh leaves from five-leaf-stage plants were fixed overnight in 2% glutaraldehyde, mounted on specimen stubs using double-sided tape and coated with gold particles in a SEMPrep2 sputter coater (Nanotech, England, United Kingdom). The phenotype was analyzed by SEM (S-4800, Hitachi, Japan) with a secondary electron detector at high voltage (10 kV).

### DNA Isolation and PCR Amplification

Genomic DNA was extracted from fresh leaves using the cetyltrimethylammonium bromide (CTAB) method (Doyle, 1990). The concentration of each sample of genomic DNA was measured spectrophotometrically and adjusted to approximately 30 ng/μl.

Polymerase chain reaction (PCR) was carried out in a volume of 20 μl containing 2 μl of 10× PCR buffer (including Mg^2+^), 1.6 μl of dNTPs (2.5 mM each), 0.4 μl of Taq DNA polymerase (2.5 U/μl), 0.8 μl of forward primer (10 μM), 0.8 μl of reverse primer (10 μM), 2 μl of template DNA, and 10.4 μl of ddH_2_O. PCR was conducted on a Bio-Rad (United States) iCycler thermocycler as follows: initial denaturation step at 94°C for 5 min; 35 cycles at 94°C for 30 s, 55°C for 30 s, and 72°C for 45 s; and a final extension at 72°C for 5 min.

### Inheritance Analysis and Mapping Strategy

The glossy and glaucous phenotypes in plants were identified visually and recorded at the five-leaf stage when the difference in appearance could be easily distinguished. Segregation ratios of the F_2_ and BC_1_P_1_ populations were analyzed with the Chi-square (χ^2^) test. Two DNA pools were constructed by mixing equal amounts of DNA from 12 glossy green and 12 glaucous F_2_ individuals. Then, 2000 pairs of simple sequence repeat (SSR) primers in the laboratory were used to successively screen the parental lines and the DNA pools. Primers that demonstrated polymorphism between the parents were subsequently used to screen the two pools. The linkage relationship between the target gene and the polymorphic primers was confirmed in 128 F_2_ individuals using JoinMap 4.0 (Van Ooijen, 2006). Linkage between markers and the *BoWax1* gene was determined with the Kosambi mapping function, and a genetic map was constructed using MapDraw 2.1 software (Liu and Meng, 2003; Kosambi, 2016). To finely map the mutant gene, 50 pairs of new primers based on primary mapping results were developed. The new primers were used to analyze the parental lines and recombinants of 808 F_2_ recessive individuals. The sequences of all polymorphic markers used in this study are listed in **Table 1**

**Table 1:** Sequences of primers used in this study.

Primer name	Forward primer sequence (5′–3′)	Reverse primer sequence (5′–3′)
BoID000046	CACAAAATCATTAGGCCAAC	AAAAGACAGTGCCTTCCTAA
C01gSSR127	GGCGTGAGACAGTCCAAT	TGAGAACCTTGCTTACAAAC
C01gSSR129	ATGCATCATGTTCGTTACTG	TCACTGGTAAATGATGGGTT
C01gSSR132	CAAAACATGAGACCAAGACC	GCTGGATAGCATTCTAAAGG
C01gSSR135	CACAACTTATTCGCTGACAA	CACTCTTATTACGTGCTCCA
C01gSSR139	TTAAGGGATTTTGGACAGG	GAAGGATATACTGTGGTGGC
C01gSSR148	TGGCGGGGACACCCTCCAAAA	TGCACTGCGTGCTAGACTATC
C01gSSR150	AGAATTCAAGTCTTTGCGAG	AAACGAGGGTTGTTTTCTTGC
Bol013612g1	ACTAGGCATAATGTGTGCG	CACGACAGTCATCAGAAGC
Bol013612g2	GTTTGGACTTGTAGTGCTTCTG	GATTATCTATGGGTGGTAACGG
Bol013612g3	AGACGAGGTTTGTCCCTTC	CATACTCTTCTAAAGCACCCG
Bol013612g4	TATCAACACACTGGGCGTC	ACACGAACCACCTTACCTTC
Bol013612g5	GGCTCACCAGGTTCTATTCC	CCCAACTTCCCAATCAGAC
Bol013612c1	CTCGCTCATACGTACATAC	TTTAAATAGCAACATCCC
qPCR-013612	AGCCTCTCCTGAAACCATC	CGGACGAATCAACACAAG
qPCR-actin-Bol	CCTCCGTCTTGACCTTGC	GTCTCCATCTCCTGCTCGT

### Sequence and Expression Analysis of the *Bo13612* Gene

The InterPro annotations for *B. oleracea* and GO annotations for *Arabidopsis thaliana* for all the genes located within the mapped region were obtained from BRAD^1^ (Cheng et al., 2011). The expression level of the *Bo13612* gene in WT and HUAYOU2 plants was determined by qPCR. Total RNA was extracted from fresh leaves at the five-leaf stage using the EasyPure^®^ Plant RNA Kit (TRANS) as described by the manufacturer. First-strand cDNAs were synthesized in a 20-μl reaction volume containing approximately 7 μg of RNA and oligo(dT) primers using the TransScript One-Step gDNA Removal and cDNA Synthesis Kit (TRANS). Real-time PCR was performed with ABI SYBR Green on an ABI 7900HT Fast Real-Time PCR System (Applied Biosystems) according to the manufacturer’s instructions. The β-actin gene was used as an internal control (Okazaki et al., 2007). The 2^-ΔΔC_t_^ method was used to analyze the data (Livak and Schmittgen, 2001). Three technical replicates were performed in this study. The sequence of the primers for qPCR, qPCR-013612 and qPCR-actin, are listed in **Table 1**. Each sample was tested in triplicate. The sequences of the *Bo13612* gene were amplified using the gDNA and cDNA as templates with the KAPPA HiFi HotStart ReadyMix PCR Kit (Kappa Biosystems). The PCR products were sequenced by BGI TechSolutions, Co., Ltd., and the sequences were aligned using Multalin software^2^ (Corpet, 1988).

Primers used to amplify the gDNA sequence were designed using Primer Premier 5 software and date from the *B. oleracea* genome database (BRAD^1^). The primers were named Bol013612g1, Bol013612g2, Bol013612g3, Bol013612g4, and Bol013612g5 (**Table 1**) (Lalitha, 2000). The primer used to amplify the cDNA of the *Bol013612* sequence was designed based on data from the *B. oleracea* transcriptome database^3^ and was named *Bol013612*c1 (**Table 1**) (Liu et al., 2014).

## Results

### The Morphological Appearance Is Altered in HUAYOU2

**FIGURE 1:**
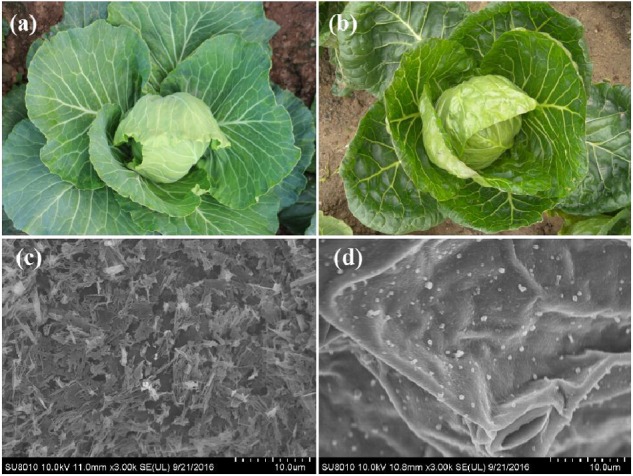
T Phenotypic characteristics of WT and HUAYOU2. **(a)** Appearance of leaf in WT; **(b)** appearance of leaf in HUAYOU2. **(c)** SEM showing wax crystals on the surface of leaves in WT; **(d)** wax crystals on the surface of leaves in HUAYOU2.

Regarding the coverage of cuticular wax, the appearance of the leaves in the WT cabbage was glaucous (**Figure 1a**). In contrast to WT, the leaves of HUAYOU2 tended to be glossy green, with little wax on the plant surface (**Figure 1b**). SEM was used to investigate the micro-characteristics and density of the wax crystals on the leaf surface. The results showed that the surface of the WT leaf is covered with compact wax crystals with a scale- and rod-like shape (**Figure 1c**). Unlike the WT, there are very few wax crystals covering the leaf surface of HUAYOU2 leaves, and these wax crystals tended to have a particle-type shaped (**Figure 1d**). This indicates that the glossy phenotype of HUAYOU2 is caused by a decrease in cuticular wax crystals, which is consistent with observations of many wax-deficient mutants in other plants (Pu et al., 2013).

### Inheritance Analysis of the Glossy Green Trait in HUAYOU2

The appearance of all 100 F_1_ plants from the crosses of HUAYOU2 and M36 was glaucous, indicating that the glaucous trait was dominant over the glossy green trait. Among 3328 F_2_ individuals, 2520 had glaucous leaves, and 808 had glossy green leaves, corresponding to a segregation ratio of 3:1 by the Chi-square test. The segregation ratio in the BC_1_P_1_ population was 1:1 (210 glaucous:222 glossy), and all the BC_1_P_2_ plants had a glaucous appearance (**Table 2**). These results demonstrated that inheritance of the glossy green trait in HUAYOU2 follows a monogenic recessive pattern.

**Table 2:** Segregation of glaucous and glossy traits and the χ^2^ goodness-of-fit test of segregation in BC_1_P_1_ and F_2_ populations.

Population	Total	Non-glossy	Glossy	Segregation ratio	χ^2^
P_1_(HUAYOU2)	20	0	20	–	–
P_2_(M36)	20	20	0	–	–
F_1_	100	100	0	–	–
F_2_	3328	2520	808	3.12:1	0.88
BC_1_P_1_(F_1_ × HUAYOU2)	432	210	222	0.95:1	0.28
BC_1_P_2_(F_1_ × M36)	200	200	0	–	–

### Fine Genetic and Physical Mapping of the *BoWax1* Gene

**FIGURE 2:**
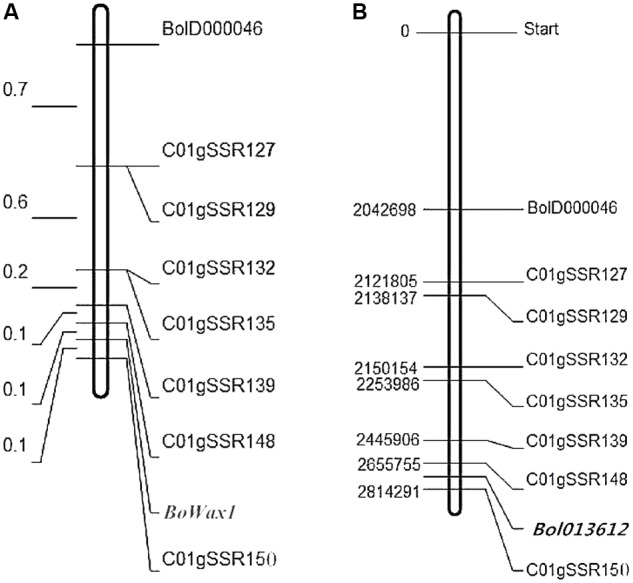
Genetic map (left, units: cM) of *BoWax1* on chromsome C01 **(A)**. The corresponding physical map positions (right, units: Mb) are also shown **(B)**.

A total of 2000 SSR markers available in the laboratory were used to identify polymorphisms in the parental lines (HUAYOU2 and M36) and in the glossy and glaucous DNA bulks. As a result, 114 pairs of primers were identified that showed polymorphism between the parental lines, but only BolD000046 was identified as being polymorphic between two DNA pools. Marker BolD000046 was mapped close to *BoWax1* using 128 segregating F_2_ population plants (**Figure 2A**).

To saturate the genetic map containing *BoWax1*, 50 pairs of primers near primer BolD000046 were designed based on the physical position of marker BolD000046. Eight of the 50 newly developed primers showed polymorphism between parental lines HUAYOU2 and M36. The eight SSRs were subsequently screened to investigate recombination using 808 F_2_ recessive individuals. Based on information from the polymorphic markers and the recombinants, the genetic distance between the two closest flanking makers, C01gSSR148 and C01gSSR150 (**Figure 2A**), was found to be 0.2 cM. The physical distance between primers C01gSSR148 and C01gSSR150 is 158.5 kb (**Figure 2B**). All of the primers are on chromosome C01 and the order of the markers on the genetic map is consistent with that on the physical map.

### Candidate Gene Analysis

**FIGURE 3:**
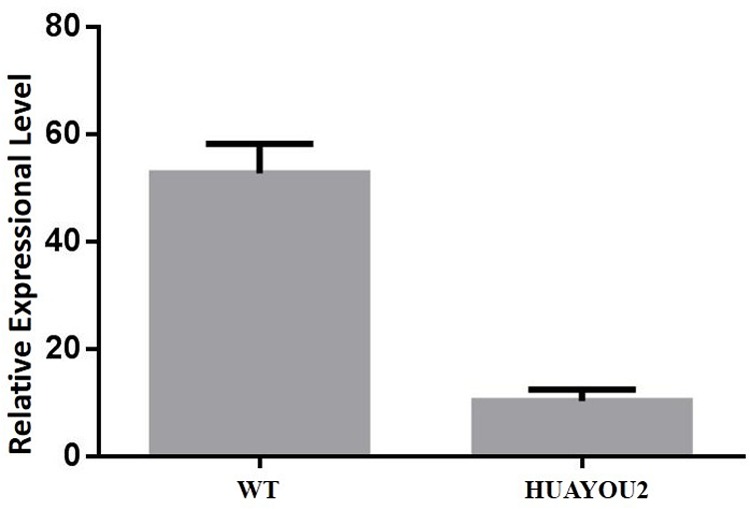
Expression level (Y axis) of gene *Bol013612* in HUAYOU2 and WT. qPCR analysis showing expression level in HUAYOU2 is significantly decreased compared with that of the WT. Standard errors are also presented.

Analysis of the genomic region between C01gSSR148 and C01gSSR150 was carried out. Gene annotations in BRAD and alignments with *A. thaliana* showed that gene *Bol013612* contains the conserved FAR domain, which plays important roles in wax biosynthesis and is present in proteins that are responsible for primary alcohols synthesis (Rowland et al., 2006). *At4g3370* is an example of an *Arabidopsis* gene that contains the conserved FAR domain and exhibits the same glossy stem wax phenotype in two mutant lines (cer4-1 and cer4-2). A deletion and/or rearrangement of the promoter region occurs in the cer4-1 mutant and a T-DNA insertion is present in the fourth intron of At4g33790 (at nucleotide 1,960 relative to the start codon) in the cer4-2 mutant (Rowland et al., 2006). According to the homologous analysis results, the *Bol013612* gene was predicted to participate in the alcohol-forming pathway. Aside from *Bol013612*, no other genes in the mapped region were found to be related to plant wax metabolism (Gene analysis and annotations are supplied in Additional file 1: Supplementary 2). The *Bol013612* gene was selected as the candidate for expression analysis using qPCR. The results showed that the expression level of *Bol013612* is significantly decreased compared with that of the WT, implying that transcription of the *Bol013612* gene was affected (**Figure 3**).

**FIGURE 4:**
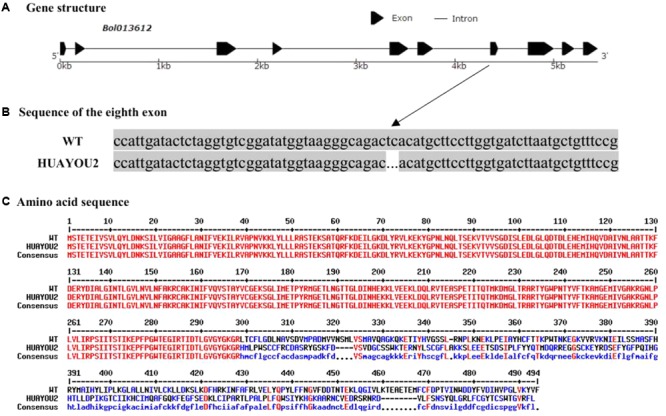
The gene structure and alleic variation in *Bol013612* **(A)**. Sequence alignment of exon 8 showing two bp deletion in HUAYOU2 as compared to WT **(B)**. Amino sequence alignments showing premature stop codon (indicated with arrow) due to a frameshift mutation in *Bol013612* gene in HUAYOU2 **(C)**.

The *Bol013612* gene was sequenced to further determine whether the glossy green trait of HUAYOU2 is caused by a mutation in *Bol013612*. The *Bol013612* gene, numbered MG808275 in NCBI database, is 5,448 bp in length, including 10 exons and 9 introns (**Figure 4A**). The The coding sequence (CDS) of *Bol013612* is 1,482 bp in length. The sequencing results showed that there was a deletion of two bases in the eighth exon of HUAYOU2 (sequences of WT and HUAYOU2 are supplied in Additional file 1: Supplementary 1). The deletion mutation was suspected of causing a frameshift mutation in the *Bol013612* gene and a premature stop codon (**Figures 4B,C**). This might disrupt the function of this gene, which may influence the wax biosynthesis and subsequently cause the glossy appearance of HUAYOU2.

## Discussion

Waxes are secreted onto the surface or into the interior of cuticles to form the first barrier against various environmental stresses (Millar et al., 1999). Since Dellaert discovered the first waxy cuticle *eceriferum* mutant (*cer*) in *A. thaliana* (Dellaert et al., 1979), many genes related to wax synthesis have been isolated, including wax synthesis-related enzymes (*CER4, CER6, CER10, FATB*, and *GL8*) (Fiebig et al., 2000; Bonaventure and Ohlrogge, 2003; Dietrich et al., 2005; Zheng et al., 2005; Rowland et al., 2006), wax transporters (*CER5* and *WBC11*) (Pighin and Samuels, 2004; Bird et al., 2007; Panikashvili et al., 2007), and transcription factors (*SHN1, MYB30, MYB96*, and *WXP1*) (Broun et al., 2004; Zhang et al., 2005; Raffaele et al., 2008; Seo et al., 2011). Several genes implicated in *Brassica* plant wax biosynthesis pathway were reported in recent studies. Though these studies identify the same glossy waxless appearance, the inheritance patterns are different. For example, the waxless trait of the Chinese cabbage mutant 08A235-2 is controlled by a single recessive gene (Zhang et al., 2013), same as that of HUAYOY2 in this study. By contrast, a dominant mutant allele controls the waxless trait in *Brassica napus* mutant 6-1025 (Pu et al., 2013). The glossy appearance of HUAYOU2 in this study is also determined by a single recessive gene, but sequence and functional analysis of the candidate gene revealed that the novel locus for glossiness on chromosome C01 is involved in the primary alcohol-forming pathway, in contrast with the gene *BnaA.GL* in *Brassica napus* (Pu et al., 2013) and the gene *BrWax1* in *Brassica rapa* (Zhang et al., 2013).

C*er4* mutant and cabbage mutant LD10GL are fertile in contrast with the cabbage glossy green mutant 10Q-961, in which only a few seeds were produced after self-pollination (Liu Z. et al., 2017). Enzymes containing the conserved FAR domain usually act on medium- and long-chain fatty acids and have been reported to be involved in the biosynthesis of plant cuticular wax (Rowland et al., 2006). In *Arabidopsis*, FAR has specificity for very-long-chain fatty acids and is present in enzymes responsible for the synthesis of primary alcohols in the epidermal cells of aerial tissues and in the roots (Rowland et al., 2006). *CER4* is one of the genes encoding an FAR domain in *Arabidopsis*. The glossy wax-deficient trait is displayed in *Arabidopsis* mutant *cer4* due to decreases in the production primary alcohols and wax esters.

By studying the cabbage mutant HUAYOU2, *BoWax1* was finely mapped and the target gene was identified. In the F_2_ population, gene *BoWax1* was localized to a region of 158.5 kb on cabbage chromosome C01. The sequence variation and abnormal expression of the *Bol013612* gene imply that *Bol013612* may be the target gene that accounts for the glossy green trait of HAUYOU2. These results will help us further elucidate the formation of the glossy trait in HUAYOU2 and accelerate wax biosynthesis studies in cabbage. More research needs to be carried out in subsequent work, such as functional studies of *BoWax1*, investigation of the metabolic mechanism of cabbage wax biosynthesis and identification of other genes involved.

## Author Contributions

DL, LY, and ZF designed and supervised the study. XD, ZZL, JT, MZ, YZ, HL, YL, and ZSL participated in its design. DL participated in the mapping, RT-PCR, and sequence analysis. JT, ZZL, YL, and ZSL participated in the statistical analysis. DL and LY wrote the manuscript. All the authors discussed the results and contributed to the manuscript. All authors read and approved the final manuscript.

## Conflict of Interest Statement

The authors declare that the research was conducted in the absence of any commercial or financial relationships that could be construed as a potential conflict of interest.

## Footnotes

1 http://brassicadb.org/brad/index.php

2 http://multalin.toulouse.inra.fr/multalin/multalin.html

3 http://www.ncbi.nlm.nih.gov/geo/query/acc.cgi?acc=GSE42891
