# Supplementary material for: Fine Mapping and Candidate Gene Identification for Wax Biosynthesis Locus, BoWax1 in Brassica oleracea L. var. capitata
Source: Front Plant Sci. 2018 Apr 30;9:309. doi: 10.3389/fpls.2018.00309 (PMC5937124; doi:10.3389/fpls.2018.00309)
Supplement: Supplementary file 1 [file Table_1.DOC]

**Supplementary 1** CDS of gene *Bol013612* in WT and HUAYOU2.

**CDS of gene *Bol013612* in WT**

ATGTCGACAGAAACGGAGATCGTGAGTGTTCTTCAGTACCTTGACAACAAATCCATATTGGTCATTGGAGCTGCTGGGTTCTTAGCAAATATTTTCGTGGAGAAGATATTAAGGGTGGCACCTAACGTGAAGAAACTCTATCTTCTTCTAAGAGCATCAACTGAAAAATCTGCTACCCAGAGGTTTAAAGACGAGATTTTAGGGAAGGACTTGTACAGGGTACTGAAGGAGAAGTATGGTCCAAATCTAAATCAACTTACATCTGAGAAAGTTACTGTTGTCAGTGGGGACATTTCCCTTGAGGATCTGGGTCTTCAAGACACTGACTTGGAACATGAGATGATCCACCAAGTTGATGCCATTGTTAATTTAGCTGCAACTACTAAATTTGATGAAAGATACGATATAGCACTTGGTATCAACACACTGGGCGTCCTCAATGTCTTGAATTTCGCCAAGAGATGTGCAAAGATTAATATTTTTGTTCAAGTATCAACAGCTTACGTTTGCGGGGAAAAATCTGGTTTGATAATGGAAACACCATACCGTATGGGTGAGACGTTGAATGGAACCACCGGCCTAGACATCAACCATGAGAAGAAATTGGTCGAGGAGAAACTTGACCAGCTCCGTGTCACCGAAGCCTCTCCTGAAACCATCACTCAAACCATGAAAGATATGGGGCTCACCAGGGCAAGAACGTATGGATGGCCAAACACTTACGTTTTCACAAAAGCAATGGGAGAGATGATTGTAGGGGCAAAAAGGGGGAATTTACCACTTGTGTTGATTCGTCCGTCAATTATTACTAGCACTATCAAAGAACCATTCCCTGGCTGGACCGAAGGCATCAGGACCATTGATACTCTAGGTGTCGGATATGGTAAGGGCAGACTCACATGCTTCCTTGGTGATCTTAATGCTGTTTCCGATGTGATGCCAGCAGATATGGTAGTAAATTCGATGTTAGTGTCGATGGCTGTTCAAGCTGGAAAACAGAAAGAAACTATTTATCATGTGGGTTCCTCGCTAAGAAATCCCTTGAAGAATGAGAAACTTCCTGAGATAGCATACCATTGTTTTACTACCAAACCATGGACTAACAAAGAAGGGAAGGTGGTTCGTGTAAAGAATATCGAGATTCTGAGTTCTATGGCTAGTTTCCACAGATACATGGCCATACATTACTTGATCCCATTAAAGGGACTTGCATTATTAAACATTGTATTATGCAAGCTTTTGGACAAAAGTTTGAAGGATTTTCATAGGAAGATAAACTTTGCATTCCGGCTCGTTGAACTTTACCAGCCCTACCTCTTTTTCAATGGAGTATTTGATGATACAAACACGGAAAAGCTGCAAGGAATTGTGTTGAAGACAGAAGCCGAAACCGAGATGTTCTGTTTTGATCCAACAGTTATCAATTGGGACGACTATTTTGTGGATATACATGTTCCTGGACTGGTTAAGTACGTTTTCTAA

**CDS of gene *Bol013612* in HUAYOU2**

ATGTCGACAGAAACGGAGATCGTGAGTGTTCTTCAGTACCTTGACAACAAATCCATATTGGTCATTGGAGCTGCTGGGTTCTTAGCAAATATTTTCGTGGAGAAGATATTAAGGGTGGCACCTAACGTGAAGAAACTCTATCTTCTTCTAAGAGCATCAACTGAAAAATCTGCTACCCAGAGGTTTAAAGACGAGATTTTAGGGAAGGACTTGTACAGGGTACTGAAGGAGAAGTATGGTCCAAATCTAAATCAACTTACATCTGAGAAAGTTACTGTTGTCAGTGGGGACATTTCCCTTGAGGATCTGGGTCTTCAAGACACTGACTTGGAACATGAGATGATCCACCAAGTTGATGCCATTGTTAATTTAGCTGCAACTACTAAATTTGATGAAAGATACGATATAGCACTTGGTATCAACACACTGGGCGTCCTCAATGTCTTGAATTTCGCCAAGAGATGTGCAAAGATTAATATTTTTGTTCAAGTATCAACAGCTTACGTTTGCGGGGAAAAATCTGGTTTGATAATGGAAACACCATACCGTATGGGTGAGACGTTGAATGGAACCACCGGCCTAGACATCAACCATGAGAAGAAATTGGTCGAGGAGAAACTTGACCAGCTCCGTGTCACCGAAGCCTCTCCTGAAACCATCACTCAAACCATGAAAGATATGGGGCTCACCAGGGCAAGAACGTATGGATGGCCAAACACTTACGTTTTCACAAAAGCAATGGGAGAGATGATTGTAGGGGCAAAAAGGGGGAATTTACCACTTGTGTTGATTCGTCCGTCAATTATTACTAGCACTATCAAAGAACCATTCCCTGGCTGGACCGAAGGCATCAGGACCATTGATACTCTAGGTGTCGGATATGGTAAGGGCAGACACATGCTTCCTTGGTGATCTTAATGCTGTTTCCGATGTGATGCCAGCAGATATGGTAGTAAATTCGATGTTAGTGTCGATGGCTGTTCAAGCTGGAAAACAGAAAGAAACTATTTATCATGTGGGTTCCTCGCTAAGAAATCCCTTGAAGAATGAGAAACTTCCTGAGATAGCATACCATTGTTTTACTACCAAACCATGGACTAACAAAGAAGGGAAGGTGGTTCGTGTAAAGAATATCGAGATTCTGAGTTCTATGGCTAGTTTCCACAGATACATGGCCATACATTACTTGATCCCATTAAAGGGACTTGCATTATTAAACATTGTATTATGCAAGCTTTTGGACAAAAGTTTGAAGGATTTTCATAGGAAGATAAACTTTGCATTCCGGCTCGTTGAACTTTACCAGCCCTACCTCTTTTTCAATGGAGTATTTGATGATACAAACACGGAAAAGCTGCAAGGAATTGTGTTGAAGACAGAAGCCGAAACCGAGATGTTCTGTTTTGATCCAACAGTTATCAATTGGGACGACTATTTTGTGGATATACATGTTCCTGGACTGGTTAAGTACGTTTTCTAA

Sequence alignment of CDS of gene *Bol013612* in WT and HUAYOU2


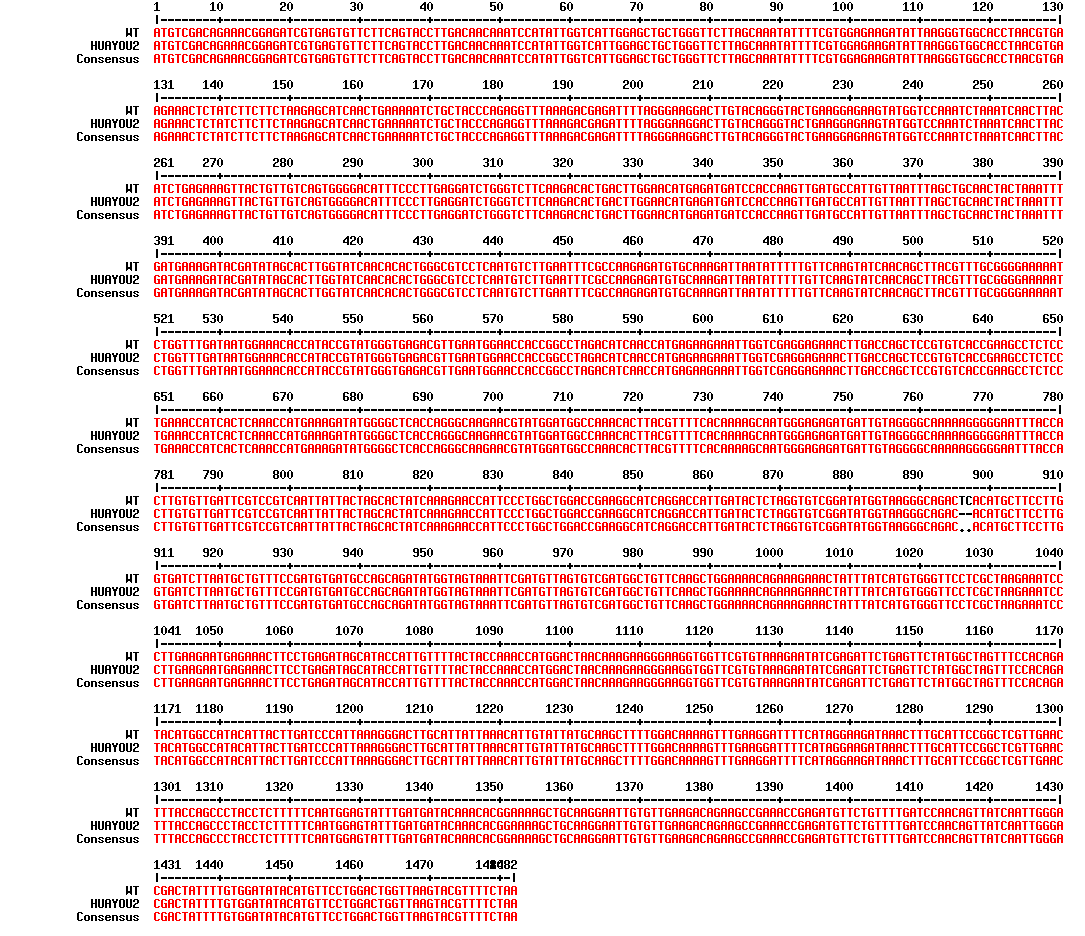


**Supplementary 2 Annotation of the *B. oleracea* genes in the candidate region**

| Gene ID^a^ | Gene position on C01 ^b^ | | Function Annotation ^c^ | *A. T.* ID ^d^ | *A. T.* annotations ^e^ |
| --- | --- | --- | --- | --- | --- |
| [Bol013575](http://119.97.203.210/cgi-bin/bolbase/gene_detail.cgi?locus=Bol013575) | 2749341 | 2751647 | EPI1_CAEEL Laminin-like protein | AT4G33390 | Plant protein of unknown function (DUF827) |
| [Bol013576](http://119.97.203.210/cgi-bin/bolbase/gene_detail.cgi?locus=Bol013576) | 2746644 | 2749008 | Quinoprotein amine dehydrogenase | AT4G33400 | dem protein-related / defective embryo and meristems protein-related |
| [Bol013577](http://119.97.203.210/cgi-bin/bolbase/gene_detail.cgi?locus=Bol013577) | 2743181 | 2744486 | Peptidase A22B | AT4G33410 | signal peptide peptidase family protein |
| [Bol013578](http://119.97.203.210/cgi-bin/bolbase/gene_detail.cgi?locus=Bol013578) | 2739879 | 2741535 | Haem peroxidase | AT4G33420 | peroxidase, putative |
| [Bol013579](http://119.97.203.210/cgi-bin/bolbase/gene_detail.cgi?locus=Bol013579) | 2736322 | 2739327 | Tyrosine-protein kinase | AT4G33430 | BAK1, RKS10, SERK3, ELG, ATSERK3, ATBAK1; BAK1 (BRI1-ASSOCIATED RECEPTOR KINASE); kinase/ protein binding / protein heterodimerization/ protein serine/threonine kinase |
| [Bol013580](http://119.97.203.210/cgi-bin/bolbase/gene_detail.cgi?locus=Bol013580) | 2731841 | 2734647 | glycoside hydrolase family | AT4G33440 | glycoside hydrolase family 28 protein / polygalacturonase (pectinase) family protein |
| [Bol013581](http://119.97.203.210/cgi-bin/bolbase/gene_detail.cgi?locus=Bol013581) | 2729182 | 2730032 | Myb transcription factor | AT4G33450 | ATMYB69, MYB69; MYB69 (MYB DOMAIN PROTEIN 69); DNA binding / transcription activator/ transcription factor |
| [Bol013582](http://119.97.203.210/cgi-bin/bolbase/gene_detail.cgi?locus=Bol013582) | 2724886 | 2726490 | ABC transporter | AT4G33460 | ATNAP13, EMB2751; ATNAP13; transporter |
| [Bol013583](http://119.97.203.210/cgi-bin/bolbase/gene_detail.cgi?locus=Bol013583) | 2723809 | 2724168 | uncharacterized | AT4G33467 | unknown protein |
| [Bol013584](http://119.97.203.210/cgi-bin/bolbase/gene_detail.cgi?locus=Bol013584) | 2720720 | 2723191 | Histone deacetylase superfamily | AT4G33470 | hda14, ATHDA14; hda14 (histone deacetylase 14); histone deacetylase |
| [Bol013585](http://119.97.203.210/cgi-bin/bolbase/gene_detail.cgi?locus=Bol013585) | 2718363 | 2720303 | uncharacterized | AT4G33480 | unknown protein |
| [Bol013586](http://119.97.203.210/cgi-bin/bolbase/gene_detail.cgi?locus=Bol013586) | 2714653 | 2716507 | Peptidase aspartic, active site | AT4G33490 | aspartic-type endopeptidase |
| [Bol013587](http://119.97.203.210/cgi-bin/bolbase/gene_detail.cgi?locus=Bol013587) | 2712978 | 2714216 | Peptidase C12, ubiquitin carboxyl-terminal hydrolase 1 | AT4G33495 | RPD1; RPD1 (ROOT PRIMORDIUM DEFECTIVE 1); ubiquitin thiolesterase |
| [Bol013588](http://119.97.203.210/cgi-bin/bolbase/gene_detail.cgi?locus=Bol013588) | 2708632 | 2712219 | Protein phosphatase | AT4G33500 | protein phosphatase 2C-related / PP2C-related |
| [Bol013589](http://119.97.203.210/cgi-bin/bolbase/gene_detail.cgi?locus=Bol013589) | 2703472 | 2706167 | DAHP synthetase | AT4G33510 | DHS2; DHS2 (3-deoxy-d-arabino-heptulosonate 7-phosphate synthase); 3-deoxy-7-phosphoheptulonate synthase |
| [Bol013590](http://119.97.203.210/cgi-bin/bolbase/gene_detail.cgi?locus=Bol013590) | 2697711 | 2702624 | ATPase | AT4G33520 | PAA1, HMA6; PAA1 (P-TYPE ATP-ASE 1); ATPase, coupled to transmembrane movement of ions, phosphorylative mechanism / copper ion transmembrane transporter |
| [Bol013591](http://119.97.203.210/cgi-bin/bolbase/gene_detail.cgi?locus=Bol013591) | 2693541 | 2697080 | K+ potassium transporter | AT4G33530 | KUP5; KUP5; potassium ion transmembrane transporter |
| [Bol013592](http://119.97.203.210/cgi-bin/bolbase/gene_detail.cgi?locus=Bol013592) | 2690194 | 2692550 | Beta-lactamase-like | AT4G33540 | metallo-beta-lactamase family protein |
| [Bol013593](http://119.97.203.210/cgi-bin/bolbase/gene_detail.cgi?locus=Bol013593) | 2688125 | 2689621 | Carbonic anhydrase | AT4G33580 | ATBCA5, BCA5; carbonic anhydrase family protein / carbonate dehydratase family protein |
| [Bol013594](http://119.97.203.210/cgi-bin/bolbase/gene_detail.cgi?locus=Bol013594) | 2685806 | 2686234 | uncharacterized | AT4G33585 | unknown protein |
| [Bol013595](http://119.97.203.210/cgi-bin/bolbase/gene_detail.cgi?locus=Bol013595) | 2681854 | 2685346 | GH3 auxin-responsive promoter | AT5G51470 | auxin-responsive GH3 family protein |
| [Bol013596](http://119.97.203.210/cgi-bin/bolbase/gene_detail.cgi?locus=Bol013596) | 2672510 | 2677841 | SUMO deconjugating cysteine peptidase | AT4G33620 | Ulp1 protease family protein |
| [Bol013597](http://119.97.203.210/cgi-bin/bolbase/gene_detail.cgi?locus=Bol013597) | 2670937 | 2672122 | uncharacterized | AT4G33625 | unknown protein |
| [Bol013598](http://119.97.203.210/cgi-bin/bolbase/gene_detail.cgi?locus=Bol013598) | 2669821 | 2670279 | uncharacterized | AT4G33640 | unknown protein |
| [Bol013599](http://119.97.203.210/cgi-bin/bolbase/gene_detail.cgi?locus=Bol013599) | 2664247 | 2669263 | GTPase effector domain | AT4G33650 | ADL2, DRP3A; DRP3A (DYNAMIN-RELATED PROTEIN 3A); GTP binding / GTPase/ phosphoinositide binding |
| [Bol013600](http://119.97.203.210/cgi-bin/bolbase/gene_detail.cgi?locus=Bol013600) | 2662821 | 2663233 | uncharacterized | AT4G33660 | unknown protein |
| [Bol013601](http://119.97.203.210/cgi-bin/bolbase/gene_detail.cgi?locus=Bol013601) | 2661312 | 2661563 | uncharacterized | AT4G33666 | unknown protein |
| [Bol013602](http://119.97.203.210/cgi-bin/bolbase/gene_detail.cgi?locus=Bol013602) | 2657896 | 2660820 | Pyridoxal phosphate-dependent transferase | AT4G33680 | AGD2; AGD2 (ABERRANT GROWTH AND DEATH 2); L,L-diaminopimelate aminotransferase/ transaminase |
| [Bol013603](http://119.97.203.210/cgi-bin/bolbase/gene_detail.cgi?locus=Bol013603) | 2655982 | 2657264 | uncharacterized | AT4G33690 | unknown protein |
| [Bol013604](http://119.97.203.210/cgi-bin/bolbase/gene_detail.cgi?locus=Bol013604) | 2652992 | 2655617 | uncharacterized | AT4G33700 | CBS domain-containing protein |
| [Bol013605](http://119.97.203.210/cgi-bin/bolbase/gene_detail.cgi?locus=Bol013605) | 2650723 | 2651330 | ATPase | AT3G21180 | ACA9, ATACA9; ACA9 (AUTOINHIBITED CA(2+)-ATPASE 9); calcium-transporting ATPase/ calmodulin binding |
| [Bol013606](http://119.97.203.210/cgi-bin/bolbase/gene_detail.cgi?locus=Bol013606) | 2648269 | 2648793 | Allergen V5\/Tpx-1 related | AT4G33720 | pathogenesis-related protein, putative |
| [Bol013607](http://119.97.203.210/cgi-bin/bolbase/gene_detail.cgi?locus=Bol013607) | 2640354 | 2640872 | uncharacterized | AT4G33730 | pathogenesis-related protein, putative |
| Bol013608 | 2636946 | 2638274 | uncharacterized | AT4G33740 | unknown protein |
| Bol013609 | 2632755 | 2636138 | Aspartyl-tRNA synthetase | AT4G33760 | tRNA synthetase class II (D, K and N) family protein |
| Bol013610 | 2629852 | 2632103 | magnesium ion binding | AT4G33770 | inositol 1,3,4-trisphosphate 5/6-kinase family protein |
| Bol013611 | 2617108 | 2619581 | uncharacterized | AT4G33780 | SHW1 |
| Bol013612 | 2609971 | 2615418 | catalytic activity | AT4G33790 | CER4, G7, FAR3; CER4 (ECERIFERUM 4); fatty acyl-CoA reductase (alcohol-forming)/ oxidoreductase, acting on the CH-CH group of donors |
| Bol013613 | 2594539 | 2594539 | uncharacterized | AT4G33800 | unknown protein |
| Bol013614 | 2590724 | 2592041 | peroxidase activity | [AT4G33870](javascript:modalDialog('multiSearchAt.php?gene=AT4G33870','select database',390,200)) | peroxidase, putative |

^a^ Genes in the candidate region.

^b^ Physical position of the seventeen *B. oleracea* genes on chromosome C01.

^c^ Swissprot annotations for the *B. oleracea* genes obtained from BRAD.

^d^ The best hits of genes from *B.oleracea* to *A.thaliana* (AT).

^e^ GO annotations for the seventeen genes from *B. oleracea* to *Arabidopsis thaliana* obtained from BRAD.
